# Health literacy in Beijing: an assessment of adults’ knowledge and skills regarding communicable diseases

**DOI:** 10.1186/s12889-015-2151-1

**Published:** 2015-08-19

**Authors:** Daitao Zhang, Shuangsheng Wu, Yi Zhang, Peng Yang, C. Raina MacIntyre, Holly Seale, Quanyi Wang

**Affiliations:** Beijing Center for Disease Prevention and Control, No.16 He Pingli Middle Street, Dongcheng District, Beijing, 100013 China; School of Public Health and Community Medicine, UNSW Medicine, The University of New South Wales, Sydney, Australia

## Abstract

**Background:**

There have been a number of studies conducted to date looking at the issue of health literacy, but none have been conducted in Beijing, China. The aim of this study was to evaluate the communicable diseases health literacy (CDHL) levels of Beijing residents towards three key areas: knowledge, adoption of preventative measures/behaviours, and health skills.

**Methods:**

A structured survey was undertaken with Beijing residents aged ≥18 years. A multistage stratified sampling approach was used to identify and recruit residents. Participants were excluded if they were foreigners, residents of Hong Kong, Macau or Taiwan, or were unable to communicate in Mandarin.

**Results:**

The questionnaire was completed by 11052 participants, with a moderate accuracy rate (average: 61.3 %) and a good discrimination level (average: 0.428). Cronbach’s alpha was 0.748. The items were grouped into three subscales representing knowledge, adoption of preventative measures and behaviours, and health skills. Correlations of the subscales and the Total Score is significant (*P* < 0.01), and all the three subscales correlate strongly with the Total Score The mean CDHL score of Beijing inhabitants was 15.28. The percentage of those who were identified as having adequate CDHL was 41 %.

**Conclusions:**

The total CDHL level of residents in Beijing was relatively low, particularly in those residing in the suburbs, those above 60 years of age, manual workers, and the illiterates. Gender, age-group, level of education, occupation, self-reported health status and region were all shown to be significantly predictive of CDHL. It is important that more resources are dedicated to improving the CDHL in Beijing, given the risk of emerging and re-emerging infectious diseases in the region.

## Background

The concept of health literacy is an important but complex concept, and since it was first propositioned there has been continuing debate about the definition and the approaches used to measure the levels [1–5]. The World Health Organization (WHO) defines health literacy simply as “an individual’s ability to gain access to understand and use health information” for promoting and maintaining health [6, 7], referring to the definition of health literacy given by the National Assessment of Adult Literacy, the Institute of Medicine Committee for Health Literacy and the American Medical Association (AMA) [8].

People with low health literacy tend to adopt fewer preventive services and less health information technology. As a result, they use emergency department more frequently, have poorer health outcomes and a higher risk of death [9–12]. It is particularly urgent to identify these community groups and to take measures to improve their CDHL.

There are a number of approaches which can be utilised to measure the “degree to which individuals and communities have the capacity to obtain, process, and understand basic health information and services needed to make appropriate health decisions” [13]. These include the Rapid Estimate of Adult Literacy in Medicine (REALM) [14], the Test of Functional Health Literacy in Adults (TOFHLA) [15], the Shortened Rapid Estimate of Adult Literacy in Medicine (REALM-R), the Shortened Test of Functional Health Literacy in Adults (S-TOFHLA), the Newest Vital Sign (NVS), the eHealth Literacy Scale (eHEALS), the Health Literacy Screening Question Methodologies (HLSQM) [16]. Generally, the TOFHLA and REALM tests are regarded as the gold standards for assessing the health literacy of patients [16]. The TOFHLA is used to measure patients comprehension towards health information and characterizes them as having adequate, marginal or inadequate health literacy [15]. The REALM test is the most commonly used health literacy assessment tool in the clinical setting. It tests the ability of patients to read and pronounce common medical terms [17].

While the morbidity and mortality associated with communicable diseases has been steadily declining for Beijing residents, new threats have continued to emerge in the last ten years including SARs and influenza H5N1 and H7N9 [18–23]. In order to formulate appropriate prevention/control and communication strategies it is important to understand the level of CDHL level of Beijing residents. Therefore, this new study aimed to evaluate CDHL levels of Beijing residents towards three key areas: knowledge, adoption of preventative measures/behaviours, and health skills. The distinction between the three areas is based on the health literacy definition of the WHO [6, 7].

## Methods

### Instrument development

The study aimed to measure and assesses health literacy. Items for the questionnaire were identified following a review of the published research. Chinese studies that explored the knowledge attitude, and practices of participants related to infectious diseases prevention and control were reviewed for possible variables. In addition, variables from the National Health Literacy Survey [24–27] that measured residents’ level of basic knowledge, adoption of preventative measures and behaviors, or health skills regarding common communicable diseases were extracted. In order to identify the most contextually appropriate variables, only studies conducted in Mainland China were utilized.

The items that were chosen for inclusion were discussed and evaluated by a small group of professionals, including three clinical doctors and three public health experts from the field of infectious diseases treatment and prevention. The three clinical doctors worked in the infectious diseases department of an infectious diseases hospital, a tertiary hospital and a secondary hospital within the Beijing region respectively, and the three public health experts were from Beijing Center for Diseases Prevention and Control (CDC), a district CDC office, and a community health center. Following the panel discussion, a total of 25 items were chosen for inclusion. The final questionnaire consisted of three sections that measured: knowledge (8 items), adoption of preventative measures and behaviors (12 items), and health skills (5 items).

The final survey was available in Chinese and took five to ten minutes to be completed. Values were assigned to each of the questions: (1) one point for a correct or positive answer; and (2) zero points if the answer to the question was incorrect or negative. For the item “How many days did you do physical activities more than 30 min per day in the past one week?” one point was assigned if participants answered “≥3 days”.

### Subjects and survey design

A multistage stratified sampling approach was used to recruit participants. In 2008, The National Health Literacy Survey showed the expected percentage of people with adequate health literacy was 21.82 % among city residents. The percentage was represented by symbol π and the numerical value was 0.2182 [17]. The sample size of each subgroup was calculated by the function *n* = μ_α_^2^ × π(1-π)/δ^2^ × deff. The confidence interval (CI) was ±10 %, maximum permissible error (δ) =0.1π, μ_α_ = 1.96, and the design effect of complex sampling (deff) = 1.5, which was used to adjust the effectiveness loss of complex sampling instead of random sampling. Therefore the minimum sample size of each subgroup was 2,065. Considering the differences among different age groups (18–29, 30–39, 40–49, 50–59 and 60+), a total of five subgroups were identified. The total sample size of the five subgroups was expected to be 10,325 (2,065/subgroup × 5). Taking into consideration the response rates and efficiency rates of the questionnaire, the actual sample size was increased by 5 % to a total of 10,841.

There are 16 districts in Beijing, which are divided into urban and suburban districts based on population density. The population density was >6548 people per km^2^ in the urban districts and ≤ 1305.4 people per km^2^ in the suburban districts. Six districts including three urban districts and three suburban districts were chosen to be sampled. Five towns or streets were randomly selected in each district, and five resident committees or villages were randomly selected in each town or street. In total, 150 residents’ committees or villages were confirmed as survey locations. In every location, about 73 residents were randomly selected as subjects based on the name lists of residents acquired from residents’ committees. Participants were resident’s aged ≥18 years that were able and willing to give their informed consent to participate and who had continuously lived in Beijing for more than half a year. Participants were excluded if they were foreigners, residents of Hong Kong, Macau or Taiwan, or were unable to communicate in Mandarin.

### Data collection

The survey was conducted between December 2010 and January 2011. Participants were asked to complete the questionnaire by themselves or with the help of trained study staff if they had difficulty with reading or writing.

### Statistical analysis

The database was established in Epidata 3.1, and analyzed using SPSS 11.5. Based on the score distribution, the average score of participants whose percentages were the closest to the top 25 % and bottom 25 % seperately were treated as the two cut points. The discrimination index (D) was calculated by taking the average score of the top 23.8 % of participants (PH, score ≥ 19) minus that of the bottom 22.9 % (PL, score ≤ 12). Reliability was calculated by internal consistency measures, using Cronbach’s alpha formula. Inter-scale correlations were calculated by the means of Pearson’s correlation coeffcients. Confirmation factor analyses were conducted to verify the scale’s construct validity. Confirmatory factor analysis was implemented by the Amos 7. Cut-point of adequate health literacy was determined via receiver operating characteristic (ROC) analyses based on their educational level. Frequencies were calculated for categorical variables. One-way ANOVA was used to compare the differences of health literacy scores between subgroups. The Pearson *χ*^2^ test was used to compare the different proportions of respondents with CDHL in different groups. Multivariate unconditional logistic regression analysis was conducted to determine factors associated with the CDHL level. The variables with *P* < 0.10 in univariate analysis were included in multivariate analysis. Backward logistic regression was conducted by removing variables with *P* > 0.10. Statistical significance was defined as *P* < 0.05.

### Ethics approval

The study protocol was approved by the Research Ethic Committee of Beijing Center for Disease Prevention and Control.

## Results

### Demographic characteristics

The questionnaires were distributed to13287 Beijing residents, and 11052 of them completed the survey, with a response rate of 83.2 %. The demographic characteristics of the residents is included in Table 1.

**Table 1:** Health literacy score and Percentage of adequate health literacy by Participant characteristics

Participant characteristics		Number	Health literacy score			Percentage of adequate health literacy (score > =17)		
			Mean	Std.	P	n	%	*P*
Gender								
	male	5344	15.1	4.11	<0.001	2112	39.5	0.002
	female	5701	15.44	4.02		2417	42.4	
Nationality								
	Han majority	10246	15.28	4.09	0.582	4219	41.2	0.244
	The minority	806	15.36	3.76		315	39.1	
Age group								
	18–29	2239	15.90	3.82	<0.001	1042	46.5	<0.001
	30–39	2180	16.09	3.85		1069	49	
	40–49	2195	15.53	3.68		910	41.5	
	50–59	2278	15.02	4.08		870	38.2	
	60-	2160	13.86	4.49		643	29.8	
Highest level of education								
	illiteracy	295	9.88	4.16	<0.001	17	5.8	<0.001
	primary school	1084	12.28	4.08		162	14.9	
	junior high school	3008	14.34	3.77		867	28.8	
	senior high school	3194	15.71	3.63		1390	43.5	
	college or above	3462	17.12	3.47		2098	60.6	
Occupation								
	students	378	15.86	3.96	<0.001	190	50.3	<0.001
	peasants	2973	13.81	4.14		779	26.2	
	manual workers	289	13.94	3.97		75	26	
	employee of enterprise	1888	16.53	3.49		1008	53.4	
	migrant workers	325	14.49	4.05		104	32	
	service workers	887	15.26	3.79		355	40	
	civil servants and public institutions’ staff	1116	16.92	3.59		652	58.4	
	healthcare workers	303	18.96	3.25		236	77.9	
	retirees	1854	15.32	3.91		747	40.3	
	people waiting for employment	826	14.33	4.10		265	32.1	
	others	213	16.46	3.45		123	57.7	
Self-reported health status								
	excellent	2478	16.26	3.86	<0.001	1279	51.6	<0.001
	good	3980	15.59	3.92		1728	43.4	
	normal	4009	14.71	4.02		1374	34.3	
	bad	503	12.94	4.72		132	26.2	
	very bad	78	12.83	4.39		18	23.1	
Region								
	urban	5729	15.97	3.90	<0.001	2767	48.3	<0.001
	suburb	5323	14.54	4.12		1767	33.2	
Overall		11052	15.28	4.07		4534	41	

### Reliability and validity of CDHL

The average percentage of correct answer was 61.3 %. The overall discrimination index was 0.428. Most of the items selected were answered with a moderate accuracy rate (average: 61.3 %) and with a good discrimination index (average: 0.428) (Table 2). Cronbach’s alpha was 0.748, indicating acceptable internal consistency for all of the items. Based on confirmatory factor analysis, the items were grouped into three subscales representing knowledge, adoption of preventative measures and behaviors, and health skills (GFI = 0.95, TLI = 0.81, AGFI = 0.94, RMSEA = 0.046). (GFI, goodness-of-fit index; TLI, Tucker-Lewis index; AGFI, adjusted goodness-of-fit index; RMSEA, root mean square error of approximation). For further testing of the dimensionality of CDHL, correlations of the subscales and the Total Score were examined by means of Pearson correlation coefficient. Correlations of the subscales and the Total Score is significant (*P* < 0.01), and all the three subscales correlate strongly with the Total Score (Table 3).

**Table 2:** Percentage of correct answer, discrimination index, and item-total correlation of health literacy items

	Items	% correct	PH	PL	D	Item-total correlation
Knowledge	What is the normal body temperature?	36.3	0.221	0.523	0.302	0.232
	What diseases do vaccines protect against?	86.6	0.702	0.984	0.283	0.367
	Which is the best way for influenza prevention?	79.1	0.574	0.952	0.378	0.371
	Which is the best way for measles prevention?	46.5	0.286	0.681	0.395	0.287
	What are the typical clinical symptoms of tuberculosis?	74.7	0.540	0.943	0.403	0.377
	How is hepatitis A spread?	48.6	0.293	0.731	0.438	0.327
	Which of the following is the correct path for the transmission of hepatitis B?	23.9	0.078	0.512	0.433	0.360
	Which of the following is the correct path for the transmission of HIV?	39.6	0.138	0.698	0.560	0.416
Preventative measures and behaviors	How many days did you do physical activities more than 30 min per day in the past week?	38.1	0.280	0.498	0.218	0.173
	Do you spit up phlegm in public?	93.9	0.818	0.996	0.178	0.337
	Do you cover up when you want to sneeze or cough?	93.4	0.806	0.994	0.189	0.343
	Do you wash your hands before eating, after using the bathroom?	54.4	0.258	0.840	0.582	0.445
	Do you wear mask when visiting someone at the hospital?	20.9	0.063	0.420	0.357	0.322
	Do you often open the windows to keep	86.4	0.688	0.977	0.289	0.348
	The air circulation during the respiratory infectious diseases epidemics?					
	Do you separate raw and cooked food when cooking or preserving them?	77.2	0.549	0.942	0.393	0.375
	Will you visit restaurants with poor sanitation?	28.8	0.109	0.568	0.459	0.371
	Do you share towels with others?	66.6	0.410	0.886	0.476	0.391
	Do you visit a dentist at an irregular outpatient clinic?	92.4	0.832	0.988	0.156	0.263
	Do you uptake rabies vaccine after a dog or cat bite?	76.1	0.530	0.938	0.408	0.379
	Will you pay attention to infectious disease epidemics at your destination when you want to travel?	72.8	0.421	0.935	0.514	0.458
Health skills	Can you easily get the information about infectious diseases?	41.2	0.091	0.817	0.725	0.534
	Can you read drug instructions?	48.1	0.115	0.889	0.773	0.562
	Can you easily read popular science readings about infectious diseases?	48.3	0.096	0.916	0.820	0.598
	Can you read laboratory sheets?	47.9	0.190	0.818	0.628	0.468
	Can you use the thermometer?	76.9	0.579	0.927	0.348	0.341
Overall		61.3	0.387	0.815	0.428

**Table 3:** Inter-Scale Correlations (Pearson’s correlation)

	Knowledge	Preventative measures and behaviors	Health skills	Total score
Knowledge	1^a^	0.312^a^	0.326^a^	0.692^a^
Preventative measures and behaviors	0.312^a^	1^a^	0.439^a^	0.797^a^
Health skills	0.326^a^	0.439^a^	1.000	0.746^a^
Total score	0.692^a^	0.797^a^	0.746^a^	1.000

^a^Correlation is significant at the 0.01 level

### ROC analysis

An ROC analysis was used to classify the levels of CDHL. The ROC curve is a graph of sensitivity (y-axis) versus 1 – specificity (x-axis). Maximizing sensitivity corresponds to some large y value on the ROC curve. Maximizing specificity corresponds to a small x value on the ROC curve. Thus a good first choice for a test cutoff value is that value which corresponds to a point on the ROC curve nearest to the upper left corner of the ROC graph. In this study, comparisons by education level indicated a cut-point of 16.5 for differentiating respondents with college education versus no college education (area under the curve =0.69, sensitivity = 0.61, specificity = 0.68). Based on the analysis, participants were classified into two groups: inadequate health literacy (score ≤ 16), and adequate health literacy (score ≥ 17).

### CDHL level description

The mean CDHL score for participants was 15.28. The percentage of those who were identified as having adequate CDHL was 41 %. The percentage with adequate CDHL was significantly higher in female (42.4 %) than that in male (39.5 %) (*P* = 0.002). The percentage also increased with higher education levels. Participants aged between 30 and 39 years recorded the highest percentage of adequate CDHL (49 %), by contrast, the percentage of residents aged >60 was the lowest (29.8 %). Urban residents (48.3 %) were significantly more likely to have adequate health knowledge compared to participants residing in suburban areas (*P* < 0.001) (Table 1).

### Factors associated with overall CDHL

The following factors were found to significantly impact the levels of CDHL according to the multiple logistic regression: gender, age-group, highest level of education, occupation, and self-reported health status and region. Females were more likely to have adequate CDHL (OR = 1.24, *P* < 0.001). By age group, residents aged 30–39 (OR = 1.39, *P* < 0.001), 40–49 (OR = 1.26, *P* = 0.001) and 50–59 (OR = 1.32, *P* < 0.001) possessed significantly higher level of CDHL when compared to the participant’s aged 18–29. Residents aged more than 60 years old also showed higher CDHL level, however, the difference was not statistical significant. Not surprising, the level of CDHL increased with increases in self-reported health status. Among different occupations, health workers possessed the highest CDHL level (OR = 3.13, *P* < 0.001), while manual workers have the lowest CDHL level (OR = 0.60, *P* = 0.005). The CDHL level lowered with the decreasing of self-reported health status. The residents lived in the suburb possessed significantly lower CDHL level than those who lived in the urban areas (Table 4).

**Table 4:** Multiple logistic regressions for the factors related to CDHL

Participant characteristics		OR	95.0 % C.I.for OR		*P*
			Lower	Upper	
Gender					
	male	reference			
	female	1.24	1.14	1.34	<0.001
Age-group					<0.001
	18–29	reference			<0.001
	30–39	1.39	1.22	1.59	<0.001
	40–49	1.26	1.10	1.44	0.001
	50–59	1.32	1.14	1.53	<0.001
	60-	1.19	0.99	1.42	0.059
Highest level of education					
	illiteracy	reference			<0.001
	primary school	2.74	1.63	4.61	<0.001
	junior high school	6.07	3.67	10.05	<0.001
	senior high school	10.90	6.56	18.11	<0.001
	college or above	19.18	11.47	32.07	<0.001
Occupation					
	students	reference			<0.001
	peasants	0.93	0.72	1.21	0.592
	manual workers	0.60	0.42	0.86	0.005
	employee of enterprise	1.09	0.86	1.38	0.485
	migrant workers	0.73	0.52	1.01	0.061
	service workers	0.85	0.65	1.10	0.218
	civil servants and public institutions’ staff	1.20	0.93	1.54	0.169
	healthcare workers	3.13	2.20	4.46	<0.001
	retirees	1.26	0.96	1.65	0.091
	people waiting for employment	0.82	0.62	1.08	0.160
	others	1.53	1.07	2.19	0.020
Self-reported health status					
	excellent	reference			<0.001
	good	0.65	0.59	0.73	<0.001
	normal	0.52	0.47	0.59	<0.001
	bad	0.52	0.41	0.66	<0.001
	very bad	0.46	0.26	0.81	0.007
Region					
	urban	reference			
	suburb	0.87	0.79	0.97	0.010

## Discussion

Numerous studies examining the issue of health literacy have been conducted since the concept was first introduced [28–31]. In 2007, the National Health Literacy Survey was launched to investigate the levels of health related knowledge and behaviors of Chinese residents [32, 17]. In addition, Xinyin Sun undertook a study measuring the health literacy of infectious respiratory diseases in Beijing [33]. However, most studies conducted to date have failed to cover all three elements of health literacy. We are the first group to explore the issue of health literacy amongst residents of Beijing.

From our results, we found that the proportion of Beijing residents with adequate CDHL (49.0 %) was comparatively higher when compared to the national average of 13.64 % [17]. Rates were particularly low amongst participants: (1) residing in the suburbs; (2) aged 60 years and above; (3) who were mainly engaged in manual labor, and (4) who had completed less than five years of education.

Results from our study showed that gender, age-group, highest level of education, occupation, self-reported health status and region were factors related to the CDHL level significantly. Large-scale surveys of adult health literacy have previously reported associations between at-risk groups and low general literacy skills [34].

People with lower education level demonstrate lower health literacy in comparison with people with higher education level [35, 4, 36]. Low health literacy may be a barrier in access to health information and health care medication use and the prevention of disease [4]. Therefore, low health literacy has been associated with a range of poor health outcomes [37, 38, 35]. Consequently, improving the education level of residents is a key factor in improving CDHL. At the present time, multiple intervention strategies should be applied to residents of different education levels, including timely and accurate delivery of information to the public during a disease outbreak, and additional description of who is at risk, the nature of that risk, and what can be done to avoid exposure and manage illness [39].

The percentage of adequate CDHL of those who were over 60 years old was the lowest of all age subgroups in this study (Table 1). Several factors may affect CDHL levels in those over 60. The first is the decline of memory and verbal fluency, which are strongly associated with health literacy [40]. The second is that lower education levels in those over 60 years old might limit their abilities to obtain knowledge of CDHL. In a cross-sectional survey conducted by Li Wu in China, the proportion of the elderly who had six years or more of schooling was only 19.0 % [41]. The third factor is that the decline of cognition which has an obvious and direct impact on reading comprehension may impose restrictions on acquiring knowledge of communicable disease prevention and control from mass medias [42, 43].

The percentage of adequate CDHL of residents in suburbs was significantly lower than those of residents in urban areas (Table 1). This might be related to their economic status, the allocation of medical resources and the accessibility of CDHL information. Generally, the economic level and education resources in the suburbs were lower and less than in the urban areas in China. It is well established that poverty will directly affect peoples’ ability to make “good” decisions, including those related to their health [44]. Meanwhile less education resources will limit the education level of the local residents. To date, health education and promotion efforts have mainly been launched in urban areas. To change this situation, the publicity of control and prevention knowledge of communicable diseases should be given intensively in the suburbs considering the local epidemic characteristics.

Our study has a number of key strengths. Firstly, in order to improve the practical applicability and tolerance of the subjects, the questionnaire was professionally administered in five to ten minutes. It demonstrated good internal consistency and yielded comparable results. Secondly, the majority of previous investigations have usually focused on assessing the health literacy in limited fields [9, 33], or of certain populations [45, 46]. While our study involved a range of participants from different areas. The research findings were more representative of the overall CDHL in Beijing.

Along with the concept of eHealth literacy has been developed [47], some specific skills to seeking and understanding information of health are increasingly important. However, they may not be familiar to the elders. Consequently, some traditionnal channels, such as television programs, advice from doctors and suggestions from friends remain the main avenues for the elders to improve their health literacy [48], which limits them to acquire health related knowledge as easily as young people do.

## Conclusions

Although the method adopted in our study might be different from other studies, it supplied a hint that the CDHL level of residents in Beijing was relatively low compared with those of other cities [49]. In view of the factors that lead to the low CDHL in elder people, measures aiming at improving their CDHL should be carried out. The health and healthcare related information of communicable diseases to the elder adults must be designed beyond vocabulary simplification and specifically to limit the demand on memory and verbal fluency more comprehensively. Meanwhile, public health workers need to consider the possibility of cognitive dysfunction in the elder adults, and adapt their information giving accordingly through multiple medium.

## Abbreviations

CDHL: Communicable diseases health literacy
WHO: World Health Organization
AMA: American Medical Association
TOFHLA: the Test of Functional Health Literacy in Adults
REALM-R: the Shortened Rapid Estimate of Adult Literacy in Medicine
S-TOFHLA: the Shortened Test of Functional Health Literacy in Adults
NVS: the Newest Vital Sign
eHEALS: the eHealth Literacy Scale
HLSQM: the Health Literacy Screening Question Methodologies
CDC: Diseases Prevention and Control
ROC: Receiver operating characteristic
GFI: Goodness-of-fit index
TLI: Tucker-Lewis index
AGFI: Adjusted goodness-of-fit index
RMSEA: Root mean square error of approximation
